# African Swine Fever Modified Live Vaccine Candidates: Transitioning from Discovery to Product Development through Harmonized Standards and Guidelines

**DOI:** 10.3390/v14122619

**Published:** 2022-11-24

**Authors:** David A. Brake

**Affiliations:** BioQuest Associates, LLC, P.O. Box 787, Stowe, VT 05672, USA; bioquest802@gmail.com

**Keywords:** African Swine Fever, harmonized guidelines, modified-live vaccine, regulatory, standards, World Organisation for Animal Health

## Abstract

The recent centennial anniversary of R.E. Montgomery’s seminal published description of “a form of swine fever” disease transmitted from wild African pigs to European domestic pigs is a call to action to accelerate African Swine Fever (ASF) vaccine research and development. ASF modified live virus (MLV) first-generation gene deleted vaccine candidates currently offer the most promise to meet international and national guidelines and regulatory requirements for veterinary product licensure and market authorization. A major, rate-limiting impediment to the acceleration of current as well as future vaccine candidates into regulatory development is the absence of internationally harmonized standards for assessing vaccine purity, potency, safety, and efficacy. This review summarizes the asymmetrical landscape of peer-reviewed published literature on ASF MLV vaccine approaches and lead candidates, primarily studied to date in the research laboratory in proof-of-concept or early feasibility clinical safety and efficacy studies. Initial recommendations are offered toward eventual consensus of international harmonized guidelines and standards for ASF MLV vaccine purity, potency, safety, and efficacy. To help ensure the successful regulatory development and approval of ASF MLV first generation vaccines by national regulatory associated government agencies, the World Organisation for Animal Health (WOAH) establishment and publication of harmonized international guidelines is paramount.

## 1. Introduction

African Swine Fever (ASF) virus was first isolated by R.E. Montgomery [1] and was followed a decade later by his description of “a form of swine fever” and peracute disease transmitted from wild African pigs to European domestic pigs imported into East Africa [2]. The recent centennial anniversary of Montgomery’s seminal publication is a call to action to develop a set of internationally harmonized, adopted standards and guidelines to accelerate ASF modified-live virus (MLV) first-generation vaccine research and development, regulatory approval, and market authorization.

Following the first documented ASF intercontinental movement from Africa to the Iberian Peninsula in the last 1950s, efforts to date to develop highly safe and efficacious vaccines against this high consequence transboundary animal disease have been remarkably challenging and largely ineffectual. The difficulties in ASF vaccine research and development are complex but primarily associated with knowledge gaps in our current understanding of ASF virology, immunology, and perhaps socio-economic factors. From a virology perspective, ASFV has a largely uncharacterized 170–190 kilobase pair double-stranded DNA complex genome comprised of approximately 150–170 open reading frames (ORFs), depending on the p72 genotype (>24; 4 geographical clades) and serogroup (≥8) [3]. Many of the ASFV known proteins are thought to be functionally and/or comparatively associated with host range, viral virulence, or immunomodulatory properties; however, ~50% of the ASFV ORFs lack any known function [4]. This gap significantly increases the challenges associated with the rationale engineering of ASF MLV recombinant gene deleted vaccines. There are a relatively small number of government and academic laboratories with top tier ASF vaccine expertise, and global funding levels for ASF vaccine research and development have been historical relatively low and intermittent. Lab-specific funded vaccine research approaches and interests have been largely driven by what can be accomplished (tasks) with available resources, rather than what can be collectively achieved (safe and effective ASF vaccines) by a highly collaborative global consortium of ASF virologists, molecular biologists, immunologists, vaccinologists, and clinical veterinarians. Despite these obstacles and the largely unrestrained, expeditious spread of ASF across four continents (Africa, Asia, Europe, and N. America [Hispaniola]), substantial progress has been made in ASF MLV first generation vaccine research and development as exemplified most recently by the first regulatory approved worldwide by the Vietnamese Ministry of Agriculture and Rural Development of a ASF recombinant MLV vaccine (ASFV-G-ΔI177L) [5].

Arguably, the highest singular impediment to the acceleration and transition of current and future ASF MLV vaccine discovery candidates into regulatory development is the absence of international standard guidelines for the assessment of ASF vaccine purity, potency, safety, and efficacy. This gap has led to difficulties in the interpretation and assessment of ASF MLV vaccine candidate safety and efficacy studies reported in peer-reviewed publications, and comparison of results amongst the leading global ASF vaccine laboratories.

This review summarizes the asymmetrical landscape of peer-reviewed historical and contemporary published literature on ASF vaccine discovery for domestic pigs and wild swine. To ensure ultimate regulatory success and ASF MLV first generation vaccine approvals by national regulatory and market authorization agencies, concomitant, near-term establishment of World Organisation for Animal Health (WOAH) harmonized guidelines for fit-for-purpose ASF MLV first generation gene-deleted vaccines is essential. This review offers initial recommendations to consider for the development of international standards and harmonized guidelines for purity, potency, safety, and efficacy for ASF MLV first generation vaccines specific for genotype II panzootic strains currently circulating in Europe, Asia, Africa, and Hispaniola.

## 2. Summary of Current ASF Vaccine Approaches

Since the early 1960′s studies have conclusively demonstrated susceptible pigs that survive and recover from acute ASF following experimental exposure to highly or moderate virulent wild-type ASFV or live attenuated (modified-live) ASFV, develop resistance against subsequent experimental challenge with homologous or closely related ASFV strains [6,7,8,9,10,11,12,13]. Collectively, these and similar results suggest that safe and efficacious ASF MLV first generation vaccines tailored to the genotype II panzootic strains, can be developed to meet the veterinary vaccine regulatory requirements for product licensure, market authorization and use as a prevention and control tool in European and Asian countries where ASF is currently enzootic. All ASF vaccine candidates with acceptable safety and efficacy target product profiles likely require the induction of finely orchestrated host innate, humoral, and cellular immune responses. Conventional (inactivated, naturally isolated or laboratory cell passaged) and recombinant-based (vectored subunit, DNA, rationally engineered gene-deleted modified live virus) approaches are actively being pursued. These approaches and the recent review of ASF vaccine lead candidates have been summarized by global ASF experts elsewhere [14,15,16,17,18,19,20,21,22,23,24].

The sections below briefly summarize the ASF conventional and recombinant-based vaccine approaches and are followed by a deeper dive into several of the most promising ASF MLV first-generation gene deleted vaccine candidates against the current genotype II panzootic strains associated with the Georgia 2007 ASFV lineage. These and future vaccine candidates should be carefully evaluated in the context of specific, uniform international standards: (i) purity [manufacturing], (ii) potency [manufacturing], (iii) safety [clinical, analytical] and (iv) efficacy [clinical, analytical].

### 2.1. Inactivated ASFV Experimental Vaccines

Historical attempts to produce inactivated ASFV experimental vaccines using heated ASFV-infected tissues were first described by Montgomery over a century ago [1]. Chemical inactivation using beta-propiolactone (BPL) led to a similar, largely unsuccessful outcome [6]. ASFV-infected tissues and cell extracts inactivated with BPL or other alkylating compounds admixed with Freund’s Complete Adjuvant, alum, or silica adjuvants, confer overall poor host resistance against acute clinical disease following intramuscular (IM) virus challenge 2–4 weeks post-vaccination [25]. Two doses of ASFV-infected detergent inactivated spleen preparations can confer protection [26]. However, ASFV-infected glutaraldehyde-fixed macrophages and single dose immunization fails to induce protection [27]. The chemical inactivation approach was re-evaluated several years ago using experimental vaccines comprised of binary ethyleneimine [BEI] inactivated ASFV-infected extracts prepared from infected primary cell cultures admixed with commercially acceptable oil-in-water/T-cell immunostimulant or co-polymer adjuvants. Immunized pigs received an IM direct challenge using an ASFV representative of the current genotype II pandemic strain [28]. A more recent study utilized experimental vaccines prepared with lower BEI concentrations, at higher hemagglutinin dose 50% (HAD50) vaccine doses, and formulated in other commercially acceptable adjuvants, using a 2-dose immunization route (IM-Intradermal) [29]. All these experimental vaccines fail to protect immunized pigs against ASF acute disease following IM (homologous) challenge with a genotype II challenge strain. ASFV non-chemical inactivation approaches, such as gamma-irradiation [30,31] or UV-irradiation [32] also fail to induce homologous protection following 2-dose IM immunization regimen and IM direct, challenge.

In general, whole virus inactivated adjuvanted vaccines for other infectious diseases predominantly generate low to moderate short-lived antigen specific CD4+ T helper cells but are less efficient compared to MLV in inducing CD8+ functional T cells [33]. Thus, non-replicating, inactivated ASFV vaccines are unlikely to generate durable cell-mediated protective immunity. In contrast, many MLV vaccines induce robust pathogen-specific neutralizing antibodies as well as CD4+ helper and CD8+ cytotoxic T cells thought to be important in viral clearance [34,35,36]. There is a general knowledge gap on the early protective immune mechanisms directly mediating resistance following ASF MLV vaccine immunization [37]. A seminal study by Oura et al. [38] provides direct evidence for the critical role of CD8+ cells in protection against ASFV. In historical studies, sera obtained from protected animals provides passive immunity to naive pigs subsequently challenged with ASFV, suggesting a protective role of B cells and antibodies [39,40,41].

Based on the inactivated ASF experimental vaccine studies published to date, the current consensus of leading ASF vaccine key opinion leaders is that classical virus inactivation approaches for ASFV remain an untenable strategy at the present time.

### 2.2. Recombinant Subunit ASF Experimental Vaccines (Protein-Based, DNA, Viral-Vectored or Combinations Thereof)

Recombinant, subunit vaccine approaches offer some advantages over traditional inactivated and MLV vaccine approaches. Protein based subunit vaccines typically have improved safety profiles and protein antigen delivery and/or vector systems can be selectively optimized to improve vaccine immunogenicity and efficacy. More specifically for ASF, this approach removes high biocontainment manufacturing facility considerations and preferable use of regulatory acceptable cell line(s) that can stably propagate ASF wild type virus or MLVs to sufficient viral titers necessary to meet commercially acceptable cost-of-goods. Indeed, a few recombinant subunit swine vaccines such as Classical Swine Fever [42,43,44] and porcine circovirus [45] have been successfully developed and approved by regulatory authorities. Unlike ASFV, the host protective viral antigens and protective immune mechanisms for these two swine viruses have been clearly delineated. Two significant, unsolved gaps restricting the acceleration of ASF recombinant subunit vaccine approaches are identification of (i) ASF viral protein protective antigens and (ii) definitive immune correlates of immunization host protection associated with limiting ASFV acute infection, replication, immunopathogenesis and virus long-term persistence.

Empirical and rationale approaches have been implemented in attempts to identify key sets of ASFV protective genes/antigens with conserved serogroup antigenicity. A recent ASFV subunit vaccine review provides an excellent overview of the various recombinant protein, DNA- and virus vectored-based ASF experimental vaccine approaches evaluated in proof-of-concept clinical studies [15]. Broadly speaking, the ASFV principal gene targets selected for these approaches have been the B- and T-cell immunogenic major structural protein families (e.g., viral proteins comprising or associated with the complex viral envelope and capsid shell), nonstructural proteins associated with viral replication and assembly; and a few proteins of predicted but unknown function.

The ASFV hemagglutinin, CD2v, is the first ASFV recombinant protein reported to confer protection against ASFV (E75, genotype I) low dose challenge [46]. Although highly significant at the time, this study, and subsequent studies [47,48] used proof-of-concept clinical study designs (e.g., relatively high vaccine doses, multiple immunizations, unsafe adjuvants, ASFV low challenge doses) impractical for direct transition to a regulatory product development program. Importantly, a study design to replicate these findings using CD2v based on a different ASFV strain (Pr4) shows no protection against clinical disease [49]. One or more of the sets of most common ASFV subunit proteins that have been selected for proof-of-concept efficacy studies, may require the presence of virally encoded molecular chaperone proteins for proper protein folding into physical, stable conformation(s) mimicking native structures (i.e., icosahedral capsids) present on the ASFV virion surface. For example, the major capsid protein p72 requires a virus encoded chaperone (B602L) for trimer assembly in the native virus particle [50,51]. To date, ASFV recombinant subunit protein designs have largely failed to take into consideration these biologically relevant details which may increase the future probability of success. ASFV recombinant subunit protein-based vaccine approaches require a more basic understanding of ASFV structural protein assembly and the roles of nonstructural viral proteins in this process. Attempts to use DNA plasmids encoding for ASFV genotype I structural proteins and multiple immunizations fail to confer any [52] or limited partial homologous protection [53] against acute ASF and death. A subsequent promising report using expression library, two dose DNA immunization is the first study to demonstrate > 50% protection against ASFV genotype I lethal challenge [54]. Collectively, these three studies point to the induction of ASFV-specific CD8+ T cells being important in DNA-based immune protection. DNA prime and viral-vectored boost [55] or recombinant protein boost [56] strategies are immunogenic but fail to confer protection against ASFV strains representative of the current genotype II pandemic strain. Interestingly, DNA prime—recombinant protein boosted immunized pigs show enhanced pathology and acceleration of clinical signs, viremia, and death [56]. Hypothesized antibody-mediated disease enhancement disease has also reported with some ASFV inactivated vaccines and other ASFV recombinant prime-boost systems.

Replication deficient, viral vectored prime-boost approaches have been recently reviewed in detail elsewhere [57]. Two adenovirus prime-boost studies, the first in domestic pigs using IM challenge with Georgia/07 [58], and the second in wild boars using an Armenia 07 animal seeder exposure infection model [59] show no protection. A comprehensive, highly informative study using an adenovirus prime-modified vaccinia Ankara (MVA) boost strategy incorporating 18 different ASFV genes, induces ASFV-specific T and B-cell immune responses, and reduces clinical signs and viremia in a subset of inbred and outbred immunized pigs following ASFV genotype I challenge [60]. This experimental vaccine and immunization strategy did not identify a consistent correlation between the number of interferon (IFN)γ-secreting cells and reduced viremia. The same group conducted a follow up study using an adenovirus prime—MVA boost comprised of a pool of eight virally vectored genotype I ASFV genes [B602L, B646L (p72), CP204L (p30), E183L (p54), E199L, EP153R (C-type lectin), F317L and MGF505-5R and show 100% protection [61]. Despite this highly encouraging clinical outcome, there was a no detectable correlate(s) of immune protection. Similar prime-boost strategies could be considered using a set of homologous genes from either the genotype II pandemic strain or from a virulent, contemporary genotype I African field isolate.

In conclusion, the general approaches of using various recombinant protein-based, DNA, and viral-vectored vaccine delivery platforms, or combinations thereof to identify ASFV protective antigens has been largely unsuccessful to date (Table 1).

These results are not unexpected given the difficulty in concomitant testing of two unknowns (e.g., ASFV potential protective antigens and a vaccine platform delivery system) particularly in the absence of any natural or artificial (e.g., experimental vaccine induced) correlates of adaptive immune protection.

### 2.3. Modified-Live Viruses (MLV)

Introduction. A comprehensive review of the current state of ASF vaccine approaches by ASF vaccine key opinion leaders has coalesced around ASF MLV (also known as live attenuated virus [LAVs]) approaches for first generation vaccine candidates as holding the most near-term potential [62]. ASF MLV firstgeneration vaccine candidates against the current pandemic strain are thought to offer the most promise to meet the regulatory requirements (e.g., European Medicines Agency [EMA], United States Department of Agriculture [USDA] Center for Veterinary Biologics [CVB], other national agencies) for full or conditional product licensure, or emergency use authorization (e.g., EMA/CVMP/IWP/251947/2021, USDA 9CFR 106.1). Notably, in 2022 the first ASF MLV recombinant vaccine, ASFV-G-ΔI177L [63] (see below) to satisfactorily complete the regulatory requirements for product licensure and market authorization was approved [5,64,65]. A relatively small subset of ASF MLV first generation gene deleted vaccine candidates with reasonable vaccine development potential have been described (see below). It is generally accepted that an avirulent ASF MLV is distinguished from a wild-type, virulent ASFV based on 100% survival and the absence of observed clinical signs associated with peracute, acute, subacute, or chronic ASF. This essential MLV vaccine safety characteristic may be the observed outcome following (i) an inherent genetic deficiency in the ability to efficiently replicate in the host, (ii) the ability of host innate and adaptive immune mechanisms to control viral replication and limit tissue dissemination rapidly and effectively, or (iii) a combination of both. In the context of vaccinology and ASF MLV firstgeneration vaccines, and as first postulated 65 years ago [66], it remains unclear if ASF MLV induced host resistance against virulent ‘homologous’ challenge is driven by the induction of active innate and adaptive immune protective mechanisms such as cell-mediated immunity and circulating antibodies—or by MLV persistence perhaps through viral interference. Animal virus interference, first described in 1935 [67,68] and originally referred to as a ‘state of temporary immunity’, is the observation whereby infection with an initial virus (i.e., avirulent ASF MLV inoculum) limits infection and replication of a second infecting virus (i.e., wild type virulent ASFV exposure). MLV persistence and potential viral interference has not been actively studied in ASF vaccine research using naturally isolated LAVs, recombinant MLVs, or wild type viruses that differ in phenotypic virulence (highly, moderate, and low), p72 genotype, hemagglutinin inhibition (HAI) serogroup, etc. In indirect support for ASF MLV immunization driving host resistance through active innate and adaptive immunity, if the protective mechanism is based on virus persistence or virus replication interference, then broader protection against different ASFV isolates following MLV immunization would be expected. In perhaps a long ignored 60 year old study, Malmquist concludes that a few pigs do develop true, sterile immunity following immunization with a cell passaged partially modified (attenuated) ASFV [69]. Although the molecular tools to detect small amounts of MLV that may have persisted in the tissues of these immunized pigs were unavailable six decades ago, Malmquist’s finding argues against the hypothesis that ASF MLVs utilize viral interference or long-term persistent infection mechanisms to establish host resistance.

#### Naturally Attenuated Viruses

Below is a summary of naturally attenuated ASF viruses evaluated in proof-of-concept/feasibility safety and efficacy studies with result outcomes described in the context of ASF MLV vaccine regulatory development.

NH/P68

Genotype I, non-hemadsorbing low pathogenic virus isolated from a chronically infected domestic pig that shows some residual virulence (chronic lesions, fever, viremia) [70]. NH/P68 confers solid protection in IM or oronasal immunized, asymptomatic domestic pigs that receive a subsequent IM challenge with a closely related genotype I ASFV isolate [10]. However, following direct IM or oronasal administration to 25–45 kg pigs, NH/P68 is transmitted to co-mingled, contact pigs [71]. A follow-up study confirms NH/P68 residual virulence and fails to confer heterologous protection against very low dose genotype II pandemic strain (Arm07) IM challenge [72]. Importantly, differences in Arm07 heterologous protection are observed between NH/P68 grown in swine primary alveolar macrophages versus in an established cell line (COS7). Due to likely regulatory safety concerns on NH/P68 residual virulence, and potential manufacturing issues associated with NH/P68 genome stability grown on the COS7 cell line [72], there is considerable development risk in advancing NH/P68 as a vaccine candidate. Underscoring these development risks, NH/P68 first generation recombinant viruses lacking specific virulence genes have been constructed to try to improve MLV safety while retaining genotype I homologous (L60) and genotype II heterologous protection (Arm07) [72].

OUTR88/3

This genotype I non-hemadsorbing virus isolated from infected ticks confers varying degrees of protection in European and Africa indigenous pigs against related virulent (OURT88/1, L57, Benin 97/1) and moderately virulent (Malta/78) genotype I, and virulent VIII (Malawi Lil 20/1) and X (Uganda/65) genotypes [73]. Although these results are interesting, there is clear safety evidence from several studies that OURT88/3 possesses varying degrees of residual virulence (e.g., fever, joint swelling, virus persistence) depending on the dose and administration route [12,74]. The authors imply that altering the administration route, dose, or further deletion of virulence genes to produce OUTR88/3 second generation MLVs, is necessary to advance a vaccine candidate into regulatory development. Interestingly, a recent 4+ month safety study using a OURT88/3 single IM dose (10^4^ tissue culture infectious dose 50 [TCID50]) in slightly older 9–10-week-old (21–25 kg) pigs shows that OURT88/3 fails to induce any significant clinical signs [75]. This finding is illustrative of the frequent inconsistency in safety study results obtained amongst several ASF MLV vaccine candidates. This observation may be due to in part to study design differences such as animal age, MLV cell passage number, MLV quantitation method and dose, and/or other variables. In this same study, OURT88/3 immunized pigs challenged at 4+ months post-vaccination with a virulent genotype I (Benin 97/1) challenge are fully susceptible to acute disease. Like the residual virulence reported with NH/P68, OURT88/3 safety concerns in some studies coupled with current gaps on genome stability when passaged in bone marrow derived primary macrophages and/or potential manufacturing cell lines, suggests that OUTR88/3 possesses significant development risk as a MLV vaccine candidate. Consequently, OURT88/3 second generation MLVs containing specific virulence gene deletions have been produced with improved safety profiles but to date fail to retain sufficient efficacy [76].

Lv17/WB/Rie1

This genotype II non-hemadsorbing weakly virulent virus originally isolated from a naturally infected wild boar was evaluated in an initial proof-of-concept efficacy study at a very low immunizing dose (10 TCID50) [77]. Directly inoculated and contact exposed pigs develop mild, transient infections (fever, joint swelling, viremia) or subclinical disease. One Lv17/WB/Rie1 directly inoculated, and one contact exposed pig were subsequently housed with a seeder pig that was directly infected (10 TCID50) with a related virulent field strain. Both pigs previously infected with Lv17/WB/Rie1 are protected against clinical disease. In a subsequent study reported to be the first attempt to experimentally orally immunize boars against genotype II ASFV [78], a 1000-fold higher (10^4^ TCID50) immunizing dose was orally administered to nine, 3–4 mo. boar piglets that were co-housed with 3 naive boars for one month. Safety results show that the orally administered Lv17/WB/Rei1 is well tolerated with lack of any apparent disease, although immunized boars shed MLV to the naive, contact cohorts. Although this finding could help increase oral bait vaccination coverage in the field, vaccine virus shedding carries significant environment risk of wild pigs becoming ASFV persistent carriers. Two boars were then contact exposed for ~3 weeks to 4 boars that were IM inoculated with a very low (10 HAD50) dose of a virulent genotype II strain (Arm07). Efficacy results show that the orally administered Lv17/WB/Rei1 confers a high level of protection against ASF acute disease and death. Although these results are encouraging, there is a relatively low probability in the next few years of limiting ASFV current spread among wild pigs in numerous regions throughout Europe through oral bait vaccination. A Lv17/WB/Rei1 vaccine development program for wild pigs will require numerous vaccine safety studies (e.g., reversion to virulence, repeated administration), identification of a suitable manufacturing cell line, and environmental risk assessment to meet the regulatory requirements for wild pig oral bait immunization.

In conclusion, in the absence of additional genetic manipulation, these and other naturally attenuated ASFV strains, share significant safety constraints for serious consideration as MLV vaccine candidates. The main recognized benefit of naturally attenuated ASFV strains is their use as research tools to help identify correlates of immune protection that may also be more broadly shared with ASF MLV recombinant, gene-deleted vaccine candidates.

Cell Line Passage Attenuated Viruses. First reported 60 years ago [79], the long-standing empirical approach of cell culture progressive adaptation of virulent ASFV field isolates has been applied over the decades to produce attenuated ASFV field strains that exhibit an avirulent phenotype when inoculated into pigs. However, it was quicky discovered that this approach often results in virus over attenuation, such that the LAV no longer sufficiently replicates in the host and loses the ability to confer protection following wild-type ASFV challenge [69]. Conversely, cell adapted ASF MLVs that retain the ability to protect inoculated pigs against virulent experimental challenge [80] or natural field exposure [81] often retain unacceptable level of residual virulence (e.g., chronic lesions, joint swelling, and lameness). Thus, ASF vaccine experts have known for many decades that there is a fine balance between MLV safety (residual virulence, reversion to virulence) and efficacy. Like naturally isolated LAVs, some cell passaged attenuated MLVs are being tested not as vaccine candidates but rather as research tools to: (i) dissect the cellular mechanisms associated with immune protection and disease pathogenesis [13,82], (ii) characterize genome stability using serial cell passage [83], (iii) evaluate MLV tissue distribution/longevity (wild type virus persistence in immunized hosts) relative to subsequent virulent challenge [84], and (iv) conduct seroimmunotype characterization [84]. A recent review provides further detailed information on cell passage attenuated ASFVvaccine approaches explored over the past 60 years [85].

Gene-Deleted Recombinant MLVs. Over the past approximately 20 years, improved laboratory methods have created new, rationally designed ASFV recombinant MLVs through the genetic manipulation of highly, moderately, or low (e.g., natural or cell passaged attenuated) virulent strains. Approach success is largely dependent on a priori knowledge of ASFV genes shown to be associated with virus virulence in the specific context of the parental strain/genotype/serogroup. A recent paper [23] provides an excellent systematic review of peer-reviewed publications associated with the historic evolution of ASFV recombinant MLVs containing single or multiple gene deletions produced by genetic manipulation and their initial evaluation for safety and efficacy. Efforts over the last decade to improve the residual virulence safety profile of naturally attenuated MLVs such as NH/P68 [72] and OURT 88/3 [76,86] while retaining acceptable efficacy have been unsuccessful to date. Similarly, efforts to enhance the safety profile of rationally, single gene deleted first generation MLVs such as BeninΔDP148R [87] and ASFV-G-Δ9GL [88,89] by selective deletion of a second gene improves MLV safety but negatively impacts efficacy. Lastly, use of double gene deleted first generation MLVs to produce triple gene deleted second generation MLVs with enhanced safety features show a similar unsuccessful outcome so far [90].

## 3. Summary of MLV Gene Deleted Vaccine Candidates

Over the past 5 years, a relatively short list of ASF MLV gene deleted vaccine candidates has emerged that have been shown to be relatively safe and effective against genotype II panzootic strains. The published results from proof of concept and/or feasibility studies for each candidate, despite the relatively low number of total pigs evaluated to date for most of these vaccine candidates, supports their consideration for transition to regulatory development programs for more rigorous purity, potency, safety, and efficacy evaluation to meet the regulatory requirements for ASF MLV first generation vaccines.

### 3.1. Single Gene Deleted

BA71ΔCD2. The highly virulent genotype I Spanish isolate, BA71 [91] was used to produce a recombinant MLV through CD2 (EP402R) gene deletion and replacement with a positive marker β-glucuronidase reporter gene [92]. In an initial proof-of-concept safety (Table 2) and efficacy (Table 3) trial, 6–8-week-old pigs were immunized IM using a low (10^3^ plaque forming unit [PFU]) dose. No clinical signs or detectable viremia were observed over the 24-day post-immunization safety phase. Following direct IM homologous virus challenge (10^3^ HAD50); 20 lethal dose 50% [LD50]) dose, all the pigs survived without evidence of clinical signs. In a subsequent feasibility efficacy trial, pigs immunized IM at 10^3^ PFU, 3.3 × 10^4^ PFU, or 10^6^ PFU doses were similarly challenged 24 days later. All the pigs immunized at the intermediate or high dose show no significant ASF clinical signs or viremia at any time post-challenge, with partial protection against death in the lowest dose. In a third efficacy trial using heterologous genotype I virus (E75) challenge, all the intermediate and high dose immunized pigs survive with no to low viremias. In a fourth safety and efficacy trial, 33% of the immunized pigs with either 3.3 × 10^4^ PFU or 10^6^ PFU present with limit duration viremias and nasal virus shedding that resolve by 24 days post-immunization. Following heterologous genotype II virus (Georgia 07) challenge, 100% of the immunized pigs in the intermediate and high dose groups survive, and the majority (56%) of immunized pigs show no clinical signs. BA71ΔCD2 was also assessed for the ability to protect against heterologous challenge using ticks infected with RSA/11/2017 (genotype XIX, clade D) or direct challenge with Ken06.Bus (genotype IX, clade A). Five of six and two of six immunized pigs survive challenge, respectively [93]. BA71ΔCD2 genetically stable growth in the COS-1 continuous cell line offers a significant advantage over other ASF MLV first generation vaccine candidates (see below) only produced in swine primary cell culture systems. Although BA71ΔCD2 offers the potential for cross-protection (heterologous p72 genotype) efficacy, the retention of residual virulence at doses up to 10^6^ PFU requires further safety studies (e.g., reversion to virulence study ideally above the target release dose) in larger groups of pigs.

To improve on BA71ΔCD2 residual virulence observed at higher immunizing dose, recombinant viruses lacking both CD2v and a second virulence-associated gene were generated and evaluated for safety and efficacy. Results show that deletion of either DP96R or EP153R from BA71∆CD2 does not improve safety and decreases vaccine efficacy [94].

SY18ΔI226R. The ASFV-SY18 highly virulent genotype II Chinese isolate [95] was used to produce this recombinant virus through deletion of the highly conserved I226R gene known to interfere with host innate antiviral responses [96], and replacement with a positive marker reporter gene (eGFP) [97,98]. In a proof-of-concept safety (Table 2) and efficacy (Table 3) IM vaccine doses at moderate (10^4^) or relatively high (10^7^) TCID_50_ doses does not cause any clinical signs over the 3-week post-immunization safety phase. Reduced, transient viremia sporadic virus shedding (oral and rectal swabs) is reported. At 3 weeks post-immunization the high dose group IM challenged with parental ASFV-SY18 survive without evidence of clinical signs including fever. Viremia and transient virus shedding (oral, fecal) is detected during the 4–5-week post-challenge phase. All the SY18ΔI226R immunized/SY18 challenged surviving pigs were necropsied. Results show normal tissue pathology and no evidence of SY18ΔI226R or wild-type SY18 challenge viral DNA, leading the authors to conclude that this vaccine candidate confers ‘transmission-stopping immunity’. However, long term contact exposure of SY18ΔI226R immunized/SY18 challenged surviving pigs to naive pigs was not reported in this study. Future development studies will likely need to identify a suitable manufacturing cell line and completion of more rigorous safety studies, including backpassage/reversion to virulence, preferably at a higher dose (e.g., above the target release dose) than reported [98]. A more thorough evaluation of SY18ΔI226R horizontal transmission from immunized to native contacts would further strengthen the safety profile. Lastly, other labs should consider constructing and testing ΔI226R recombinant viruses from a different genotype II pandemic parental virus (e.g., Arm07, Georgia 07), or using one or more virulent genotypes representative of African ASFV strains, to determine if ΔI226R is a single gene deletion target for broader vaccine coverage.

**Table 2:** Clinical Models to Assess MLV Vaccine Candidate Safety.

MLV Vx Candidate	Breed/Age-Weight/Route	*N*	Immunizing Dose	Obs. Period (dpv)	Readouts
Lv17/WB/Rie1 [78]	N/A, 3–4 mo/10–15 kg or 6–8 wks	9	10^4^ TCID_50_	30	CS, T, V
		3	contact		
		3	10^3.6^ HAD_50_	14	
		3	10^3.3^ HAD_50_		
		3	2 doses of 10^2.6^ HAD_50_	28	
		3	10^2.8^ HAD_50_		
BA71ΔCD2 [92,93]	Landrace × Lg White, 6–8 wks., IM	6	10^3^ PFU	24	CS, T, V, NS
		6	10^3^ PFU	24	none reported
		6	3.3 × 10^4^ PFU		
		6	10^6^ PFU		
		6	10^3^ PFU	24	none reported
		6	3.3 × 10^4^ PFU		
		6	10^6^ PFU		
		6	3.3 × 10^4^ PFU	24	CS, T. V, NS
		6	10^6^ PFU		
	Lg. White, 15–30 kg, IM	6	10^6^ PFU	16	CS
		6	3.3 × 10^4^ PFU	21	CS
		6	10^6^ PFU		
		4	3.3 × 10^4^ PFU (D0/D21)	42	CS, T, VS
SY18ΔI226R [98]	Landrace, IM	5	10^4^ TCID_50_	21	CS, T, V, OS, RS
		5	10^7^ TCID_50_		
ASFV-G-Δ9GL/UK [99]	Crossbreed Yorkshire, 80–90 lb., IM	9	10^2^ HAD_50_	21	CS, T, V
		10	10^4^ HAD_50_		
		15	10^6^ HAD_50_		
ASFV-SY-18-ΔCD2v/UK [100]	Lg White × Landrace, 16–20 kg, IM	5	10^4^ TCID50	28	CS, T, V, NS
ASFV-G-ΔMGF [101]	Crossbreed Yorkshire, 80–90 lb., IM	10	10^2^ HAD_50_	28	CS, T, V
			10^4^ HAD_50_		
ASFV-G-ΔI177L/ΔLVR [102]	Crossbreed Yorkshire, 80–90 lb., IM	5	10^2^ HAD_50_	28	CS, T, V
		5	10^4^ HAD_50_		
		10	10^6^ HAD_50_		
HLJ/18-7GD [103]	SPF Large, ~50 days, IM	4	10^3^ TCID_50_	21	CS, T
		4	10^5^ TCID_50_		
		6	10^7^ TCID_50_		CS, LN, qPCR
		3/BP	blind passage		CS, TS, qPCR
		2	10^7.7^ TCID_50_	21	LN qPCR
	Local commercial farm, IM	1	10^6^ TCID_50_	4 post-farrowing	T, # of healthy, stillborn, mummified piglets
		3			
		2			

Readouts: CS = clinical signs, T = temperature, V = viremia, NS = nasal swab, OS = oral swab, RS = rectal swab, TS = tissue, LN = lymph node.

**Table 3:** Clinical Models to Assess MLV Vaccine Candidate Efficacy.

MLV Vx Candidate	*N*	Challenge Route	Challenge Dose	Challenge Strain	Challenge Timepoint (dpv)	Observation Period (dpc)	Readouts
BA71ΔCD2 [92,93]	6	IM	10^3^ HAD_50_ (20 LD50)	BA71	24	24	CS, T, V, NS
	6						CS, T, V
	6						
	6						
	6		10^4^ HAD_50_ (20 LD50)	E75			
	6						
	6						
	6		20 LD_50_	Georgia/07			CST, T, V, NS
	6						
	6	tick	12 ticks/pig	RSA/11/2017	16	20	CS
	6	IM	10^2^ HAU	Ken06.Bus	21		
	6						
	4		10^2^ HAU		42	28	CS, T, V
SY18ΔI226R [98]	5	IM	10^4^ TCID_50_	SY18	21	28	CS, T, V, OS, RS, HP, T qPCR
	5		10^2.5^ TCID_50_				
ASFV-G-Δ9GL/UK [99]	9	IM	10^3^ HAD_50_	Georgia/07	28	21	CS, T, V
	10						
	15						
	5				7		
	5				14		
	5				21		
ASFV-SY-18-ΔCD2v/UK [100]	5	IM	10^4^ TCID50	ASFV-SY18	28	21	CS.T. TGP, TqPCR
ASFV-G-ΔMGF [101]	10	IM	10^3^ HAD_50_	Georgia/07	28	21	CS, T. V
	10						
ASFV-G-ΔI177L/ΔLVR [102]	5	IM	10^3^ HAD_50_	Georgia/07	28	21	CS, T. V
	5						
HLJ/18-7GD [103]	4	IM	200 PLD_50_	HLJ/18	21	21	CS, TqPCR
	4						
	5				28		
	5				70		
	5				14		
	5	oral			21		

Readouts: CS = clinical signs, T = temperature, V = viremia, NS = nasal swab, OS = oral swab, RS = rectal swab, HP = histopathology, TqPCR = tissue PCR, TGP = tissue gross pathology.

### 3.2. Double Gene Deleted

ASFV-G-Δ9GL/ΔUK. Derived from the efficacious ASFV-G-Δ9GL predecessor virus that shows unacceptable residual virulence at higher doses [104], this double gene deleted virus [99] shows a relatively safety over a 3-week post-observation period following IM doses up to 10^6^ HAD_50_ in 80–90 lb. pigs (Table 2). High viremia titers are detected by 4 days post-vaccination (dpv) that peak 7 to 11 dpv and decrease to moderate titers by 3 weeks. ASFV-G-Δ9GL/ΔUK immunized pigs challenged 1-month post-immunization are protected against acute disease and death, although viremia is detectable in numerous immunized animals (Table 3). Notably in this same study, pigs immunized with a 10^4^ HAD_50_ dose are fully protected at 14 dpv following direct IM challenge with 10^3^ HAD_50_ of ASFV-G virulent virus. Interestingly, when a different parental virulent genotype II strain, HLJ/18 [105], is used to make a similar double gene deleted virus, HLJ/18-9GL&UK-del, this MLV is safe in 7-week-old pigs at immunizing doses up to 10^5^ TCID_50_, but at 3 weeks post-vaccination fails to confer protection against ASF acute disease and death following IM challenge with HLJ/18 at a high lethal dose (200 PLD_50_) [103]. Moving forward, ASFV-G-Δ9GL/ΔUK adaptation to a continuous cell line acceptable for manufacturing, followed by minimum immunizing (protective) dose efficacy studies and more extensive safety studies (e.g., backpassage/reversion to virulence above the target release dose) will be necessary to advance this vaccine candidate toward regulatory approval.

ASFV-SY18-∆CD2v/UK. Derived from the ASFV-SY18 highly virulent field strain [95], this recombinant MLV carries hemadsorption (CD2v) [106] and virulence (UK) [107] gene deletions replaced with two positive marker reporter genes (eGFP, dsRed) [100]. In a proof-of-concept safety and efficacy study in ~40-day-old (~16–20 kg) pigs (Table 2 and Table 3), an IM moderate dose (10^4^ TICD_50_) does not cause any clinical signs up to 28 dpv. Immunized pigs show no detectable viral DNA in blood or nasal swab samples up to 28 dpv. Following direct IM homologous, moderate dose (10^4^ TICD_50_) challenge at 28 dpv all immunized pigs show no ASF clinical signs. There is no molecular evidence of viremia except toward the end of the study where low virus shedding (nasal) is detected. All ASFV-SY18-∆CD2v/UK immunized/SY18 challenged surviving pigs were necropsied. Results show normal tissue pathology and overall, very low ASFV DNA levels attributed to the SY18 challenge virus. Conversely, a similar ∆CD2v/UK deletion made in another genotype II pandemic strain, HLJ/18, retains residual virulence [103]. Thus, like the next steps to consider for SY18ΔI226R, additional recombinant ∆CD2v/UK viruses made on different genotype II pandemic and African virulent genotype/serogroups backgrounds should be produced and rigorously evaluated for further safety, including testing in pregnant sows.

### 3.3. Multiple Gene Deleted

ASFV-G-ΔMGF. Based on the genotype II pandemic strain isolate, Georgia/2007, three members of the multigene family (MGF) MGF360 (MGF360-12L, MGF360-13L, MGF360-14L) and three members of the MGF505 (MGF505-1R, MGF505-2R and MGF505-3R) families have been deleted [101]. These two MGF families are associated with immunomodulation of innate immunity and host range specificity [108,109]. Initial proof-of-concept safety studies (Table 2) in 80- to 90-lbs pigs show that IM doses up to 10^4^ HAD_50_ appear safe during a 4-week post-immunization observation period. ASFV-G-ΔMGF immunized pigs are protected against clinical disease following IM challenge with virulent parental Georgia/2007 virus (Table 3). Most recent safety and efficacy studies conducted by an independent lab using ASFV-G-ΔMGF produced in an established (permanent) cell line (not specifically disclosed), confirms these initial studies in domestic pigs and further demonstrate efficacy following single dose oral administration in wild pigs [110].

Interestingly, a similar series of gene deletions using a different genotype II pandemic strain was used to produce the recombinant virus, HLJ/18-6GD. Although initial safety and efficacy results show a similar positive outcome to ASFV-G-ΔMGF [101,110], a reversion to virulence study shows that HLJ/18-6GD is genetically unstable based on the observation of clinical ASF in the two final passages in one pig [103]. Further studies are necessary to better understand the in vivo genetic stability of pandemic genotype II recombinant virus containing multiple deletions in the MGF family.

ASFV-G-ΔI177L/ΔLVR. This virus is derived from ASFV-G-ΔI177L, the first regulatory approved ASF vaccine (see below). The MLV was developed in part to address current ASFV-G-ΔI177L manufacturing limitations that use swine primary macrophage cells. The stock virus was adapted to, and serial passaged seven times on the PIPEC [111] continuous cell line. In vitro results show that ASFV-G-ΔI177L/ΔLVR has a ~10.8 kilobase pair deletion in the left variable region that fully removes several MGF members (MGF360-6L, MGF300-1L, MGF300-2R, MGF300-4L, MGF360-8L, MGF360-9L, and MGF360-10L) [102]. In two initial proof-of-concept safety studies (Table 2), 80- to 90-lb. pigs received an IM inoculation of ASFV-G-ΔI177L/ΔLVR at doses up to 10^6^ HAD_50_ and monitored for 4 weeks. Immunized pigs remain clinically healthy but present with long lasting relatively low viremias, but there is no evidence of MLV shedding and transmission to naive, co-mingled cohorts. In challenge efficacy studies, (Table 3) ASFV-G-ΔI177L/ΔLVR maintains a similar protective efficacy profile as ASFV-G-ΔI177L. ASFV-G-ΔI177L/ΔLVR is the first rationally designed ASF vaccine candidate established and propagated on a continuous cell line. It remains to be determined if PIPEC is a regulatory acceptable manufacturing cell line. If not, ASFV-G-ΔI177L/ΔLVR preferably needs to be stably adapted to a regulatory acceptable manufacturing acceptable cell line and then re-evaluated for safety and efficacy.

HLJ/18-7GD. HLJ/18 virulent parental strain [105] was used to make a seven gene deleted virus (MGF505-1R, MGF505-2R, MGF505-3R, MGF360-12L, MGF360-13L, MGF360-14L, and CD2v) containing two positive marker reporter genes [103]. Proof-of-concept safety (Table 2) and efficacy results (Table 3) at two IM immunizing doses (10^3^ and 10^6^ TCID_50_) show a favorable safety profile in 7-week-old pigs over a 3-week observation period. Following IM challenge at 21 dpv with a high lethal HLJ/18 dose (200 PLD_50_), all immunized pigs survive with some pigs showing transient fever that is immunizing dose dependent. In a pilot reversion to virulence study design, HLJ/18-7GD at a relatively high starting dose (10^7^ TCID_50_) is maintained for a short period of time in some lymph nodes but fails to show any evidence of virus transmission to naive cohorts or evidence of reversion to a virulent phenotype. An efficacy study in commercial pigs using a 2-dose immunization regimen confirms the pilot study results and shows protection following oral challenge [103]. Additional studies show duration of protection lasting at least 10 weeks following a single 10^6^ TCID_50_ IM dose. Preliminary safety results in pregnant sows are also satisfactory. Future development studies are required (e.g., whole Next Generation Sequencing [NGS], determination of wild-type virus presence at longer post-challenge timepoints), preferably to include identification of a suitable manufacturing cell line to produce active ingredient for pivotal regulatory safety and efficacy studies.

## 4. ASFV-G-ΔI177L Recombinant Licensed Vaccine (NAVET-ASFVAC)

A genotype II pandemic strain, ASFV-G, was used to produce ASFV-G-ΔI177L by deletion of the highly conserved I77L gene that has a predicted immune modulator function [63], and replacement with a reporter gene (mCherry) [112]. In an initial safety (Table 4) and efficacy study (Table 5), 80 to 90 lb. pigs were IM administered low (10^2^), moderate (10^4^), or high (10^6^) HAD50 doses. Immunized pigs do not show any clinical signs over the 4-week observation period but present with low titer viremia that persists through 28 dpv. However, there is no evidence of virus shedding to naive, co-mingled cohorts.

Following direct IM homologous, low dose (10^2^ TICD_50_) challenge (28 dpv) all the pigs survive without evidence of clinical signs including fever. There is evidence of sterilizing immunity at the moderate or high dose. A subsequent field study [64] (Table 5) shows that a 10^2^ HAD_50_ immunizing IM dose of ASFV-G-ΔI177L administered to a Vietnamese pig breed confers partial protection at 2 weeks post-vaccination, and full protection at 4 weeks post-immunization following direct challenge with a Vietnamese virulent ASFV genotype II strain. Most recently, and in direct support of vaccine approval [5,65], an ASFV-G-ΔI177L minimum immunizing dose (MID) of (10^2.6^ HAD50) was shown to be safe (Table 4) in pigs 7–8 weeks of age and is not shed to co-mingled naive cohorts. In a field study in 10-week-old pigs, two IM administered MID doses (Days 0 and 21) is safe, although dissimilar to the lab study, there is evidence of virus shedding to co-mingled naive cohorts over the 7-week observation period. Importantly, a 10× overdose and reversion to virulence studies designed to meet Vietnamese Department of Animal Health, Ministry of Agriculture and Rural Development regulatory requirements for MLV veterinary vaccines are satisfactory (Table 4**)**, and NGS comparison of the ASFV-G-ΔI177L master seed virus and the virus obtain after five backpassages in swine shows no major genetic differences [65]. To extend ASFV-G-ΔI177L current label claims for use in pigs less than 10-weeks of age and/or use in pregnant sows, additional safety and efficacy studies will be necessary. Future studies should ideally compare current commercial production yields (e.g., number of doses per mL) in swine primary macrophages to one or more continuous cell line candidates to determine if current vaccine manufacturing serial volumes can be increased.

## 5. Summary of the Current Asymmetrical Landscape on ASFV-G-ΔI177L and ASF MLV First Generation Vaccine Candidates

The absence of internationally harmonized and accepted standards for assessing these ASF MLV gene deleted vaccine candidates for purity, potency, safety, and efficacy creates inherent difficulties in the comparative interpretation of peer-reviewed published results. Each ASF vaccine research group has a working set of laboratory production (manufacturing/purity related) and analytical (potency related) methods that form the basis of in vitro and in vivo vaccine candidate characterization studies. Table 6 provides a comparative summary of these important vaccine attributes for the seven ASF MLV recombinant vaccine candidates and the ASFV-G-ΔI177L Vietnam licensed vaccine for domestic pigs, and the lead oral bait vaccine candidate for wild pigs (Lv17/WB/Rie1).

A summary of these vaccine candidates and the licensed vaccine in the context of purity, potency, safety, and efficacy is described below.

### 5.1. Manufacturing (Purity)

It is evident from Table 6 that for the ASFV-G-ΔI177L licensed vaccine and the 7 MLV vaccine candidates, all but one (BA71ΔCD2) use primary cells to generate MLV active ingredient for all the safety and efficacy clinical studies. From a vaccine purity standpoint, it is imperative that each primary cell lot used for MLV vaccine manufacturing undergoes extraneous agent testing to ensure the absence of swine pathogens and other adventitious agents. ASF MLV vaccines should be tested per WOAH Chapters 1.1.9 “Tests for sterility and freedom from contamination of biological materials intended for veterinary use” [113] (Section 2.3.4) and “Minimum requirements for the production and quality control of vaccines” (Section 1.6.3) [114]. A second purity component is to ensure that the ASF MLV master seed virus does not contain any residual wild type ASFV. Differential PCR assays and possibly NGS may be required to test ASF MLV master seed virus to unequivocally confirm the absence of parental wild type ASFV.

It is well recognized that the identification of regulatory acceptable manufacturing mammalian cell lines is a major current gap in ASF vaccine development. Several leading cell line candidates (PIPEC, WSL, ZMAC-4, COS-1 [for BA71ΔCD2]) for the large-scale production of ASF MLV vaccines have been identified [115]. Further studies are required to determine if any of these continuous cell lines can meet the regulatory purity requirements to be used for master seed virus and/or master cell stock production. Compatibility with current large-scale fixed bed bioreactor or disposable manufacturing technologies also need to be determined.

### 5.2. Analytical (Potency)

Additionally, as shown in Table 6, three different assays (PFU/mL; TCID_50_/mL; HAD_50_/mL) are typically used quantitate ASF MLVs. These assays form the basis for potency assay method development and validation for vaccine product release to support ASF MLV vaccine candidate regulatory development pivotal studies. For assay standardization it would be advantageous to identify a single continuous cell line that could be utilized for any ASF MLV potency assay. The MA-104 cell line may be an ideal candidate based on its reported comparable TCID_50_ sensitivity to primary swine macrophage, and ability to detect both hemadsorbing virus (HAD50) as well as ASF MLV non-hemadsorbing strains via p30 IFA [116]. However, some ASF MLV vaccine candidates may need to be adapted to consistently replicate in certain continuous cell lines, which may preclude their use in a standardcell line-based potency release assay. For example, wild-type ASFV requires several passages in MA-104 cells of wild-type ASFV in MA-104 cells is required to consistently grow to high titers [117]. Other transformed porcine macrophage cell lines such as PIPEC, WSL, ZMAC-4 should be evaluated against the current panel of ASF MLV vaccine candidates to determine if MLV adaptation is required for stable growth and produces consistent and reproducible titers that can be measured (e.g., HAD50, TCID50 or qRT-PCR).

### 5.3. Clinical (Safety and Efficacy)

Table 2 and Table 4 provide a high-level comparative view of the different clinical models that have been used for research proof-of-concept and feasibility studies to evaluate ASF MLV vaccine candidate and ASFV-G-ΔI177L safety. ASF MLV safety is paramount and relative to the dynamic interaction between the MLV (e.g., parental strain origin, administered dose and route) and the immunized host (e.g., breed, age, health status). Moreover, balancing ASF MLV safety concerns (e.g., reversion to virulence, shed-spread and recombination [environmental], tissue persistence) while maintaining a high efficacy level is challenging, as the therapeutic index (Effective Dose 50 [ED_50_]/Lethal Dose 50 [LD_50_]) for any ASF MLV appears to be relatively small, cannot be predicted and needs to be empirically determined in pigs. ASFV-G-ΔI177L and all the lead vaccine candidates for domestic pigs are derived from genotype II/serogroup 8 Caucasus (Georgia 2007/1) or Chinese (SY18, HLJ/18) panzootic strains, except for BA71ΔCD2, which is of European genotype I origin. Most, but not all the MLVs are evaluated at escalating doses over a 3–4 log_10_ dose range. For domestic pigs, the intramuscular immunization route is primarily used. For a product candidate targeting wild pigs (Lv17/WB/Rie1), oral inoculation is used to simulate oral bait administration. All the reported domestic pig safety studies are conducted in European origin crossbreeds, although in one study a local crossbreed is used. Domestic pigs study starting age and weight ranges are typically between 6 and 10 weeks and ~16–40 kg body weight. In all cases, the post-vaccination observation period is 3–4 weeks, although 7-week observation is used in the multiple dose study that supported ASFV-G-ΔI177L regulatory approval [65]. The primary safety readouts shared across all the studies are observed clinical signs and fever (rectal temperature). In many studies, viremia (blood) and virus shedding (nasal and/or fecal swabs) are also evaluated by RT-PCR and/or live virus isolation/titration. In a few studies, co-mingling of naive pigs with immunized pigs is used to evaluate relatively short-term MLV shed and spread (transmission). To date, backpassage and reversion to virulence safety studies have been only reported only for two ASF gene-deleted recombinant viruses [65,103].

Arguably, the most important criteria for ASF MLV vaccine candidate safety assessment are: (i) acute clinical signs and fever, (ii) shed spread to naive cohorts, (iii) environmental shedding and (iv) long-term persistence/systemic side effects. To date, the evaluation of MLV long-term persistence (e.g., ~4–6 months post-vaccination), and associated chronic side effects has not been extensively evaluated or reported for ASFV-G-ΔI177L or any of the ASF MLV first generation vaccine candidates. Notably and infrequently considered, as evidenced by the absence of any published studies, is the important environmental safety issue for the potential of ASF MLV vaccine strain recombination in the field. This may be particularly pertinent in East and South Africa where numerous co-circulating genotypes have been reported [118], as well as in specific parts of Asia where genotype I viruses are now circulating [119].

Recombination frequencies vary extensively among virus families and is well documented for many RNA viruses [120]. MLV swine and poultry vaccine strain recombination with RNA field viruses such as Porcine Reproductive and Respiratory Syndrome Virus [121] and infectious bronchitis virus [122] have been reported. Double stranded DNA (dsDNA) viruses such as ASFV generally have larger genomes because of the higher fidelity of their replication enzymes. High frequency recombination can occur in some dsDNA viruses, such as the well-studied α-Herpesviruses in which homologous recombination is relatively frequent and associated with viral replication and DNA repair [123]. Vaccinia virus, another large dsDNA virus that shares some orthologous genes with ASFV, undergoes nonhomologous (end joining) recombination and new gene acquisition with relatively low frequencies to produce novel recombinant viruses [124]. The authors hypothesize that since poxvirus infection often results in high viremia levels (similar to ASFV), vaccinia virus recombination is sufficiently frequent to seed a small pool of novel recombinant viruses with potentially novel traits into larger populations of newly produced virus particles. The potential for ASF MLV vaccine strain recombination in the field is a research gap that may be addressed through in vitro co-infection studies using new techniques to accurately estimate recombination from NGS data [125,126].

Finally, any ASF MLV first generation vaccine will likely need to demonstrate safety above the maximum release titer or the target maximum release potency dose [127].

As an example, if an ASF MLV minimum effective dose is 10^3.0^ HAD_50,_ then the product release dose will likely be ~10^4.5^HAD_50_ (~1.5 log_10_ higher to account for loss in titer over time and assay variability) with perhaps a maximum release dose (titer) of ~10^5.0^ HAD_50_. This would mean that the regulatory development safety studies for backpassage/reversion to virulence [127,128], and one dose, overdose, and repeat dose [129] would require using ≥10^5.0^ HAD_50_ per dose, and thus an 10× overdose safety would require ≥ 10^6.0^ HAD_50_. Thus, it is important that discovery research proof-of-concept and feasibility clinical safety studies evaluate MLV vaccine candidates at target doses likely to be used as the maximum dose likely to be stated in the vaccine outline of production.

Table 3 and Table 5 provides a high-level comparative view of the different clinical models that have been used for research proof-of-concept and feasibility efficacy studies to evaluate ASF MLV vaccine candidate (Table 3) and ASFV-G-ΔI177L (Table 5). Like the ASF MLV vaccine safety studies, but more complex, MLV efficacy evaluation is relative to the active interplay between the MLV (e.g., administered dose and route), virulent challenge (virus genotype/strain dose, route), post-vaccination challenge timepoint (onset of protection, duration of protection) and the immunized host (e.g., breed, age, health status). ASF MLV vaccine efficacy is first often established using the identical or a very closely related parent virulent strain (same genotype/serogroup) from which the ASF MLV is derived. All the lead vaccine candidates listed for domestic pigs (Table 3) and ASFV-G-ΔI177L (Table 5) were evaluated using an IM direct challenge method. Historically, this challenge method in domestic pigs has been the most widely used in ASF vaccine research. In an eloquent study designed to evaluate challenge dose and route-dependent effects on ASF acute clinical disease and viral dynamics [130], four challenge routes (IM, IOP, INP and direct contact) in domestic pigs were compared to determine which route best approximates natural infection while maintaining good reproducibility. The study shows that the IM challenge route in domestic pigs (i) has the highest consistency, (ii) has the lowest required dose to establish infection, (iii) but fails to simulate natural infections. The direct contact method is also highly reproducible and best simulates natural pig-to-pig ASFV transmission, however, carries the weakness of being more expensive and does not permit precise control of challenge dose and exposure timing. For pivotal efficacy regulatory studies, a direct challenge method is most often recommended. All the efficacy studies shown in Table 3 share an approximate 3- to 4-week post-vaccination timepoint for the challenge, and a similar 3- to 4-week post-challenge observation period. However, the method for challenge dose quantitation (HAD_50_, TCID_50_, LD_50_) and dose administered varies widely. All the efficacy studies use extreme morbidity/humane endpoint euthanasia or death, clinical signs, and fever as efficacy readouts, while most efficacy studies also measure viremia and at least one route of virus shedding.

Arguably, the most important criteria for ASF MLV first generation vaccine candidate efficacy assessment are: (i) protection against severe morbidity/mortality, (ii) protection against/absence of clinical signs, and (iii) reduction of challenge virus shedding [e.g., onset, maximum titer, duration]). This third criterion is critical in the context of the need for ASF MLV vaccines to prevent disease transmission. The basic reproductive ratio (R0) is a predictive parameter associated with the average number of secondary infections produced from a single infectious event [131]. Generally, a R0 value < 1 indicates an infection will not spread in a susceptible population. Future field studies should estimate the R0 value following ASF MLV vaccination in a defined population of pigs within a defined area known to have naturally circulating ASFV.

Lastly, the need to establish an internationally harmonized ASF acute disease standardized clinical scoring system for objective evaluation of ASF MLV vaccine efficacy was identified over 10 years ago [12,132,133] yet remains to be globally implemented. A standardized clinical scoring system based on the highest value acute disease objective parameter(s) such as temperature, along with a laboratory analytical readout such as viremia and/or virus shedding (as measured by RT-PCR and/or virus titration) should help strengthen future ASF vaccine development.

### 5.4. Other ASF MLV Vaccine Attributes

Heterologous Protection. It is noteworthy that with one exception (BA71ΔCD2) the broader ‘cross protection’ efficacy for the currently licensed ASFV-G-ΔI177L vaccine and six ASF MLV leading vaccine candidates has not been thoroughly valuated. This is particularly pertinent to the 32 countries in sub-Saharan Africa where ASF is enzootic or epizootic and where all 24 known different genotypes are represented [118,134]. Essential to this issue is the current lack of acceptable definition for a ‘heterologous’ virus strain. Hemadsorption inhibition (HAI) experiments and ‘cross-protection’ experiments were initially conducted in the early 1960s and different antigenic ASFV types first recognized [69,135]. The ASF MLVs derived from European/Caucasus genotype II/serogroup 8 pandemic strains are unlikely to confer protection against other ASFV genotypes from different regions of sub-Saharan Africa. As a starting point and in the near-term, it may be useful to test ASFV-G-ΔI177L or one of the ASF MLV lead vaccine candidates to confer protection against the first genotype II ASFV isolated in Africa linked to the current pandemic strains [136].

ASF MLV Vaccine Positive and Negative (DIVA) Markers. The currently licensed ASFV-G-ΔI177L vaccine and all but one (Lv17/WB/Rie1) of the vaccine candidates described above have at least one positive marker reporter gene. Although positive markers can be advantageous in the context of monitoring ASF MLV vaccination compliance, the presence of exogenous markers may be a regulatory concern. It remains to be determined if EMA CVMP, USDA CVB, and other regulatory agencies will allow the presence of positive markers in ASF MLV first generation vaccines. To date, attempts have been unsuccessful to identify one of more negative (gene deleted) markers in the ASFV-G-ΔI177L vaccine as well as in the ASF MLV lead vaccine candidates that can be used in a serology-based test to differentiate infected from vaccinated animals (DIVA). ASF MLV DIVA vaccines will most likely need to be developed for second generation vaccines along with an increase in safety and broader protection.

Future technologies. If a set of ASFV protective antigens and clear correlates of MLV-induced immune protection can be identified, it is plausible that first and second generation ASF MLV gene-deleted recombinants vaccines will be replaced by safer, newer vaccine technologies. These include viral vectored subunit, nucleic acid-based (mRNA), and virus-like particle vaccines. Analytical design of MLV ASFV-based expression vectors (ASFV-EV) and host range restricted ASF LAV (HRR-LAV) vaccine platforms have also been suggested [22]. All these conceptual future generation vaccines are expected to have enhanced safety and efficacy profiles with rapid response manufacturing capabilities to address newly emerging ASFV virulent strains.

## 6. Recommendations to Support the Development of Harmonized Guidelines for ASF MLV First Generation Vaccines: Purity, Potency, Safety and Efficacy

The major, rate-limiting impediment to the acceleration and transition of current and future ASF MLV vaccine discovery candidates into regulatory development pathways is the absence of internationally harmonized and accepted standards for ASF vaccine purity, potency, safety, and efficacy. Based on the information presented in this review on the current state of ASF vaccine research and development, initial recommendations to consider for ASF MLV first generation vaccine product development for domestic and wild pigs are summarized below:

A. Purity and Potency (Manufacturing)
  1. Specific guidelines for MLV vaccines using primary cells for product manufacturing requires SPF pigs and donor herd pathogen monitoring. If swine primary (i.e., myeloid lineage) cells are used as the manufacturing cell substrate, current WOAH (Manual of Diagnostic Tests and Vaccines for Terrestrial Animals) [113,114], EU Pharmacopoeia 10.2/Chapter 5.2.4 [137] and USDA 9CFR regulatory guidelines [138,139,140] associated with animal sources and adventitious agent screening should be followed. Note—use of primary cells does not allow consistency with the EMA manufacturing “seed lot system” and may limit production serial volume sizes.
  2. Characterize and if possible, qualify one or more current continuous cell line lead candidates for use in ASF MLV vaccine production [115].
  3. For ease in potency release assay standardization, evaluate continuous cell lines for HAD50, TCID50, or PFU dose quantitation to replace currently used swine primary cells and to potentially decrease assay variability.
B. Safety (Domestic and Wild Pig)
  1. For each target host, standardize animal model. Where appropriate, some safety studies for MLV vaccine candidates intended use in wild boar may be conducted in domestic pigs.
  2. Use current VICH [122,129], EMA, and USDA [128] guidance documents associated with target animal safety reversion to virulence/backpassage, overdose, one dose and repeat dose tests. Most preferably, the MLV dose tested in these studies should be at or above the maximum release titer or at a titer that is above target release dose stated in the outline of production.
  3. Define the minimum criteria (e.g., absence of clinical signs including fever, viremia [onset, duration, titer], absence of MLV persistence and immunologic sequalae [chronic clinical signs], and MLV spread to naive cohorts [e.g., absence seroconversion in direct contact pigs]) to demonstrate the minimum threshold of acceptable safety. Acceptable safety should be defined in the context of vaccine fit-for-purpose use in ASF enzootic, epizootic, and disease-free countries.
C. Efficacy—Domestic Pig
  1. Standardize challenge method and challenge dose. Define a required challenge route (e.g., IM) and preferable dose range and acceptable methods for back titration quantitation.
  2. Standardize animal efficacy model and clinical scoring method. Efficacy models should be development for each of the 3 recognized ASFV virulence phenotype (high, moderate, and low) and disease forms (peracute, acute, subclinical and chronic) [141,142,143,144].
  3. Define the minimum criteria (e.g., protection against mortality, protection against/absence of highest value objective clinical signs, reduction in challenge virus viremia, etc.) to demonstrate a minimum threshold of acceptable efficacy. Acceptable efficacy could be defined in the context of vaccine fit-for-purpose use in ASF enzootic or epizootic areas.
  4. Standardize animal model for onset of protection. Define the minimum criteria to demonstrate acceptable onset of immunity (protection).
  5. Standardize animal model for long-term (≥3 months post-vaccination) protection. Define the minimum criteria to demonstrate acceptable duration of immunity (protection).
D. Efficacy—Wild Pig
  1. Use the domestic pig efficacy model (above) and minimum threshold of acceptable efficacy for studies leading up to the selection of a final vaccine candidate for wild boar studies.
  2. Standardize challenge method and challenge dose. Define a required challenge route (e.g., IM) and preferable dose and back titration quantitation method.
  3. Test vaccine stability/efficacy in oral bait formulations over a prescribed total period and over a broad temperature range.
E. Analytical (supportive safety and efficacy data sets)—For animal samples obtained during safety and efficacy studies, generate a published reference list of acceptable samples and assays to include but not limited to: ASFV isolation, ASFV quantitation (e.g., RT-PCR and virus titration), and commercially available ASFV antigen and antibody tests.
F. Other Considerations.
  1. Cross-protection (use of ‘heterologous’ challenge virus). Further research on ASFV strain diversity, and serogroup classifications in the context of the CD2v and C-type lectins is required to better understand the basis of homologous vs. ‘heterologous’ (cross-protection). First generation vaccines that target the relatively limited number of ASFV genotypes/viral lineages currently present in Europe or Asia are unlikely to demonstrate acceptable cross-protection against African epizootic and enzootic strains. Generate a consensus definition of a ‘heterologous virus’. In the near-term, in ASF MLV vaccine genotype II pandemic lineage challenge-efficacy studies consideration should be given to defining cross-protection as an ASFV strain that differs from the parental strain used to construct the MLV vaccine strain.
  2. Differentiation of Infected from Vaccinated Animal (DIVA). In some, but not all circumstances, having a DIVA test that is compatible with a specific ASF MLV licensed vaccine may be advantageous, for example during the disease recovery and eradication phases following an epizootic outbreak in a previously ASFV-free country. ASF MLV DIVA vaccines in low- and middle-income countries and regions where ASFV is enzootic are arguably not needed at the present time. DIVA serology and molecular-based strategies should be pursued for ASF MLV second and third generation vaccines:
    a. In a DIVA serology strategy, the ASF MLV DIVA vaccine is preferably accompanied by an ELISA test to distinguish wild type vs. vaccine inducted antibodies. To develop this test, one or more of the ASFV deleted genes (negative marker) needs to be thoroughly evaluated for suitable immunogenicity in non-vaccinated, infected animals of all ages (including pregnant sows) and at numerous post-infection timepoints. Efforts to date to identify a target DIVA gene for deletion in any of the ASF MLV first generation vaccine candidates have been unsuccessful.
    b. A genetic DIVA strategy is predicated on identifying genetic mismatches between the ASF MLV DIVA vaccine and the wild-type field virus. This identification is typically based on multiplex real-time PCR assays that target the p72 gene of the wild-type ASFV and the deleted gene(s) of the ASF MLV DIVA vaccine. For example, a RT-PCR differential PCR DIVA prototype test for the ASFV-G-ΔI177L license vaccine has been described.
    c. Under certain circumstances, such as the oral bait vaccination of wild pig or vaccination campaign compliance monitoring in domestic pigs, a DIVA serology based on a positive gene marker may be advantageous. Since the ASFV-G-ΔI177L licensed vaccine and all the current ASF MLV first generation vaccine candidates contain at least one reporter gene (i.e., BGal, BGus, mCherry, eGFP), development of a positive marker DIVA serology test could be considered.
  3. Fit-for-purpose vaccines. ASF MLV first generation vaccines for use in countries where ASF is presently enzootic, or epizootic should have a complementary molecular DIVA (differential PCR). ASF MLV second and third generation vaccines for use in epizootic regions of countries with established control areas and surveillance zones should have a complementary serology DIVA to one or more ASFV genes.

## 7. Conclusions

Of the five main approaches for ASF vaccines (inactivated, naturally attenuated, lab passage attenuated, recombinant subunit, and recombinant gene deleted MLV)), ASF recombinant gene deleted MLV vaccine candidates offer the best near horizon promise for first generation vaccine product licensure.

There are several reasons to be cautiously optimistic that current and future ASF MLV vaccine candidates can be accelerated and transitioned from discovery research to product development, and successfully meet the regulatory requirements for ASF MLV vaccine purity, potency, safety, and efficacy. First, the recent regulatory approval of ASFV-G-ΔI177L by the Vietnam Agriculture Ministry demonstrates that a licensing roadmap for other ASF MLV vaccine candidate using internationally accepted minimum standards is feasible. Secondly, ever-increasing knowledge of the ASFV genome [143] will undoubtedly offer new gene deletion targets to improve current ASF MLV first generation vaccine candidate safety profiles while maintaining an acceptable efficacy threshold. Thirdly, several manufacturing cell lines have been identified with the potential to stably grow ASF MLVs to sufficient titers relative to target product maximum release titers. Lastly, there is strong, collective global interest in the establishment of an internationally accepted harmonized framework of analytical, clinical, and manufacturing standards for fit-for-purpose ASF MLV first generation vaccines that offer a net positive risk-benefit ratio for commercial use in the field.

From a vaccine safety perspective, it is imperative that ASF MLV vaccinated domestic and wild pigs are shown to have negligible or manageable risk in becoming vaccine strain persistent carriers and/or persistent carriers following natural exposure to virulent field strains. This well-founded concern may require that ASF MLV first generation vaccine post-licensing field pharmacovigilance data is active collected and analyzed in real time. Ideally, an active surveillance program for the detection of new ASF viruses that may arise from MLV vaccine strain and naturally circulating wild-type virus recombination should be implemented. Pharmacovigilance should also include evaluation of ASF MLV vaccine campaigns to prevent disease transmission. Backed by an international set of harmonized guidelines for ASF MLV vaccine manufacturing (purity and potency), clinical studies (safety and efficacy) and analytical assays (safety and efficacy supporting data sets), ASF MLV first generation licensed vaccines can serve as an important tool in global ASF preparedness, response, and recovery. ASF MLV first generation licensed vaccines against the genotype II pandemic strain with demonstrated acceptable safety and efficacy in specifically defined pig age ranges (e.g., pre-weaning, post-weaning, grower, and finishing) and husbandry conditions (gilts, sows, pregnant sows) can be immediately used in ASF enzootic regions to help reduce the global threat for accidental introduction into disease-free countries. In the case of new outbreaks in current disease-free countries, ASF MLV first generation licensed vaccines could be made available under emergency or conditional use in ASF-infected control (infected, buffer) zones as well as quarantine/controlled movement zones.

## Figures and Tables

**Table 1:** Summary of Recombinant Subunit ASF Experimental Vaccines (Prime-Boost).

Prime	Boost	Result	Reference
DNA	DNA	no protection	[52]
		partial protection	[53]
	Viral vector—Vaccinia	no protection	[55]
Viral vector—Modified Vaccinia Ankara (MVA)	Viral vector—Adenovirus	no protection	[60]
		protection	[61]
Viral vector—Adenovirus	Viral vector—Adenovirus	no protection	[58,59]
DNA	Recombinant Protein	no protection	[56]

**Table 4:** ASFV-G-ΔI177L Licensed Vaccine—Safety Studies.

Clinical Model—Safety							
Breed	Age/Weight	Route	*N*	Immunizing Dose	Observation Period (dpv)	Readouts	Comments
Yorkshire (Y) crossbreed	80–90 lb	IM	10	10^2^ HAD_50_	28	CS, T	[63]
			5	10^4^ HAD_50_			
			5	10^6^ HAD_50_			
Y × Landrace (L) crossbreed	20–30 kg	IM	5	10^6^ HAD_50_	28	CS, T	[64]
			25	10^2.6^ HAD_50_		CS	
Vietnamese Mong Cai crossbreed	20–30 kg	IM	5	10^1^ HAD_50_	28	CS, T	
			5	10^2^ HAD_50_			
			5	10^3^ HAD_50_			
			5	10^4^ HAD_50_			
			20	10^2.6^ HAD_50_		CS	
Y × L crossbreed	7–8 wks.	IM	4	10^2.6^ HAD_50_	28	CS, T, V NS	[64]
		N/A	4	contacts			
	10 wks.	IM	50	10^2.6^ HAD_50_	49	CS, V	field study; 2 doses (D0, 21) [65]
		N/A	10	contacts			
	7–8 wks.	IM	10	10^2.6^ HAD_50_	28	CS, T	2 doses (D0, 14) [65]
			6	10^3.3^ HAD_50_	14		5× overdose [65]
			14	10^.3.6^ HAD_50_			10× overdose [65]
Y × L crossbreed	7–8 wks.	IM	22	10^2.6^ HAD_50_	28	CS	
Vietnamese Mong Cai crossbreed	7 wks.		25				2 doses (D0, 14) [65]
Y × L crossbreed	6–8 wks.	IM	17	10^2.6^ HAD50	28		backpassage/reversion to virulence [65]

Readouts: CS = clinical signs, T = temperature, V = viremia, NS = nasal swab.

**Table 5:** ASFV-G-ΔI177L Licensed Vaccine—Efficacy Studies.

Clinical Model—Efficacy							
*N*	Challenge Route	Challenge Dose	Challenge Strain	Challenge Timpoint (dpv)	Observation Period (dpc)	Readouts	Comments
5	IM	10^3^ HAD_50_	Georgia	28	21	CS, T, V, TqPCR	proof-of concept [63]
5							
5							
5		10^2^ HAD_50_	TTKN/ASFV/ DN/2019	28	15	CS, T, V NS	efficacy in Yorkshire × Landrace crossbreed [64]
25						CS, T	
5						CS, T, V, NS	efficacy in Vietnamese Mong Cai crossbreed [64]
5							
5							
5							
20						CS, T	
18				14	14	CS, T	efficacy in Yorkshire × Landrace and Vietnamese Mong Cai crossbreed [64]
5				21			
10				28			

Readouts: CS = clinical signs, T = temperature, V = viremia, NS = nasal swab, TqPCR = tissue qPCR.

**Table 6:** Comparison of Production (Manufacturing/Purity) and Analytical (Potency) Methods.

Primary Laboratory	MLV Vaccine Candidate	Lab Production Method (Manufacturing/Purity)	Analytical Method (Potency)	Refs.
CReSA, IRTA-UAB	BA71ΔCD2	COS-1	PFU/mL on COS-1	[92,93]
Changchun Veterinary Research Institute	SY18ΔI226R *	Pulmonary alveolar macrophages (PAMs) [ASFV, CSFV, PRRSV, PRV, PPV, PCV 1/2 free by RT-PCR]	TCID_50_/mL on PAMs	[98]
USDA ARS	ASFV-G-Δ9GL/ΔUK *	blood monocyte-derived macrophages (MDMs) from commercial breed; excludes study that used proprietary/unnamed continuous cell line [110]	HAD_50_/mL on MDMs	[99]
	ASFV-G-ΔMGF *			[101]
	ASFV-G-ΔI177L/ΔLVR *			[102]
Harbin Veterinary Research Institute	ASFV-SY18-∆CD2v/UK *	PAMs from SPF pigs	TCID_50_/mL on PAMs	[100]
	HLJ/18-7GD *			[103]
Navetco	ASFV-G-ΔI177L *	PAMs form SPF pigs	HAD_50_/mL on MDMs	[64,65]
Complutense University of Madrid; INIA-CISA	Lv17/WB/Rie1 *	blood monocyte-derived macrophages (MDMs)	TCID_50_/mL on PAMs	[78]

* Genotype II pandemic strain lineage MLV.

## Data Availability

Not applicable.
